# Differences in Hip Geometry Between Female Subjects With and Without Acute Hip Fracture: A Cross-Sectional Case-Control Study

**DOI:** 10.3389/fendo.2022.799381

**Published:** 2022-02-24

**Authors:** Ling Wang, Minghui Yang, Yandong Liu, Yufeng Ge, Shiwen Zhu, Yongbin Su, Xiaoguang Cheng, Xinbao Wu, Glen M. Blake, Klaus Engelke

**Affiliations:** ^1^Department of Radiology, Beijing Jishuitan Hospital, Beijing, China; ^2^Department of Traumatic Orthopedics, Beijing Jishuitan Hospital, Beijing, China; ^3^School of Biomedical Engineering & Imaging Sciences, King’s College London, St Thomas’ Hospital, London, United Kingdom; ^4^Department of Medicine 3, FAU University Erlangen-Nürnberg and Universitätsklinikum Erlangen, Erlangen, Germany; ^5^Institute of Medical Physics, FAU University Erlangen-Nürnberg and Universitätsklinikum Erlangen, Erlangen, Germany

**Keywords:** hip fracture, geometry, cortical thickness, volume, discrimination

## Abstract

**Background and Purpose:**

Although it is widely recognized that hip BMD is reduced in patients with hip fracture, the differences in geometrical parameters such as cortical volume and thickness between subjects with and without hip fracture are less well known.

**Materials and Methods:**

Five hundred and sixty two community-dwelling elderly women with hip CT scans were included in this cross-sectional study, of whom 236 had an acute hip fracture. 326 age matched women without hip fracture served as controls. MIAF-Femur software was used for the measurement of the intact contralateral femur in patients with hip fracture and the left femur of the controls. Integral and cortical volumes (Vols) of the total hip (TH), femoral head (FH), femoral neck (FN), trochanter (TR) and intertrochanter (IT) were analyzed. In the FH and FN the volumes were further subdivided into superior anterior (SA) and posterior (SP) as well as inferior anterior (IA) and posterior (IP) quadrants. Cortical thickness (CortThick) was determined for all sub volumes of interest (VOIs) listed above.

**Results:**

The average age of the control and fracture groups was 71.7 and 72.0 years, respectively. The fracture patients had significantly lower CortThick and Vol of all VOIs except for TRVol. In the fracture patients, cortical thickness and volume at the FN were significantly lower in all quadrants except for cortical volume of quadrant SA (p= 0.635). Hip fracture patients had smaller integral FN volume and cross-sectional area (CSA) before and after adjustment of age, height and weight. With respect to hip fracture discrimination, cortical volume performed poorer than cortical thickness across the whole proximal femur. The ratio of Cort/TrabMass (RCTM), a measure of the internal distribution of bone, performed better than cortical thickness in discriminating hip fracture risk. The highest area under curve (AUC) value of 0.805 was obtained for the model that included THCortThick, FHVol, THRCTM and FNCSA.

**Conclusion:**

There were substantial differences in total and cortical volume as well as cortical thickness between fractured and unfractured women across the proximal femur. A combination of geometric variables resulted in similar discrimination power for hip fracture risk as aBMD.

## Introduction

Hip fractures are amongst the most severe consequences of osteoporosis and are associated with high morbidity and mortality and a significant reduction in the patient’s quality of life (1). Hip fracture patients have a mortality of 20% within the first year (2) and 10 to 20% of hip fracture individuals can no longer live independently (3). Hip fracture risk depends on the integrity of the proximal femur and the likelihood of experiencing forces that exceed bone strength (4). With aging, the geometrical integrity of the hip is compromised and the risk of falling increases, resulting in older individuals having an increasing risk of hip fracture. Thus, it is important to identify individuals at high risk of fracture. While areal bone mineral density (aBMD) derived from dual X-ray absorptiometry (DXA) is the routine method to evaluate osteoporosis, studies have consistently shown that it has only moderate capability to predict hip fractures (5–11).

The cortical bone of the proximal femur has become a focus of interest leading to the increased application of hip quantitative CT (QCT) in clinical trials (12). However, few studies have assessed the association of cortical bone with hip fractures, and some of these have only applied cross-sectional slice-based cortex measurements (i.e. one slice or the average of several slices) (6, 9, 13–15) instead of 3D segmented methods. Several studies have used femoral QCT to measure bone shape, volumetric BMD distribution and cortical bone thickness (CortThick) distribution (6, 11, 14, 16–18), concluding that smaller cross-sectional area, lower trabecular vBMD and thinner cortical thickness were all associated with increased hip fracture risk. However, parameters that characterize the strength of specific sub regions of bone compartments, such as bending and buckling, up to now were mostly limited to two-dimensional assessments derived from DXA hip structural analysis (HSA) (19–21). Further, DXA HSA variables are not independent of DXA aBMD (12). Assessment of femoral geometry by the QCT MIAF-Femur application (MIAF: medical image analysis framework) and volume-based structural parameters introduced by Engelke may allow for assessment of bone strength indicators in greater detail (22). MIAF-Femur software is based on 3D segmentation of the whole proximal femur, which also allows for assessment of the femoral head *in vivo* (23).

This cross-sectional case-control study aims to explore the associations of the geometrical parameters such as cortical volume and thickness with acute hip fractures. We also aim to assess differences in femoral head size between female participants with and without hip fracture.

## Materials and Methods

### Participants

The study was conducted in accordance with the Declaration of Helsinki (as revised in 2013), approved by the institutional review board of the principal investigator’s hospital, and all participants provided their written informed consent. Five hundred and sixty two community-dwelling elderly women with hip CT scans, enrolled in the China Action on Spine and Hip Status (CASH) study, were included in the study. Two hundred and thirty six of the women had an acute hip fracture and were admitted to the Emergency Department of Orthopaedic Trauma at the Beijing Jishuitan Hospital between January 2012 and May 2016. CT scans were taken within 48 hours after fracture to minimize changes in vBMD and body composition. Inclusion and exclusion criteria of the hip fracture patients were described in detail previously (23, 24). In brief, only fully ambulatory, community-dwelling Chinese Han adults with a hip fracture resulting from low-energy trauma (falls from standing or sitting height) were included (24). Participants were excluded if they had prior or bilateral hip fractures or inability to stand or walk before their hip fracture.

Three hundred and twenty six age matched women served as controls. Exclusion criteria for the control subjects were inability to sit and stand independently or inability to walk with or without an assistive device (24). Further exclusion criteria for both groups were stroke, neurological disorders, rheumatic diseases, heart failure, severe chronic obstructive pulmonary disease and coagulation disorders, and other diseases that limited function.

### QCT Scans

Spiral hip CT scans were performed for all participants using two Toshiba Aquilion scanners (Toshiba Medical Systems Division, Tokyo, Japan). A Mindways QCT calibration phantom (Mindways Software Inc., Austin, TX, USA) was scanned with each participant, and hip QCT scans were acquired in the supine position following the usual QCT procedures. Both hips were scanned from the top of the acetabulum to 3 cm below the lesser trochanter. The scan parameters were as follows: 120 kVp, 125 mAs, 1-mm thickness, 50-cm field of view (SFOV), and 512 × 512 matrix in standard reconstruction.

### MIAF Measurements

**Figure 1:**
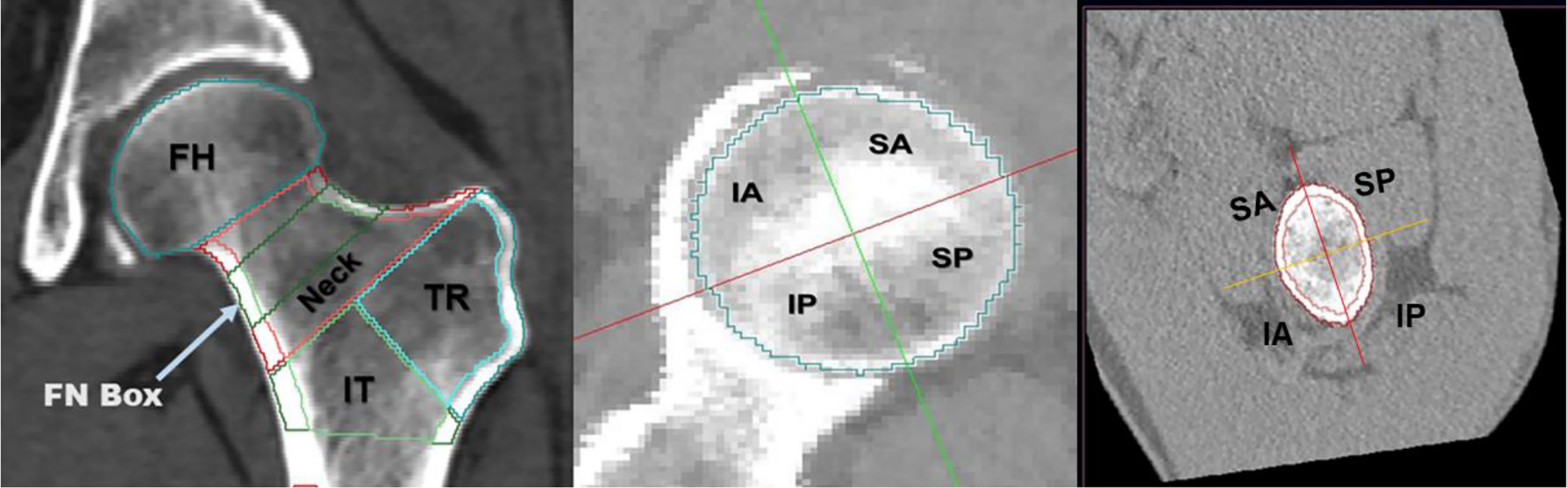
Volumes of interest (VOIs) measured at the proximal femur by MIAF-Femur (left). Axial view along with the neck axis showing anatomic quadrants of femoral head (middle) and femoral neck (right). FH, femoral head; FN, femoral neck; TR, trochanter; IT, intertrochanter; SA, supero-anterior; IA, infero-anterior; IP, infero-posterior; SP, supero-posterior.

CT images of the unfractured (hip fracture cohort) and left (control cohort) sides were analyzed by the MIAF-Femur application (Version 7.1.0MRH). The MIAF-Femur software provided standard volumes of interest (VOIs), namely the femoral head (FH), femoral neck (FN), trochanter (TR) and intertrochanter (IT) calculated relative to an anatomic coordinate system (ACS) with its origin centered at the smallest cross section of the femoral neck. The FN VOI had a height of 5 mm (**Figure 1**). The borders between VOIs were determined automatically based on anatomical landmarks and the ACS (23). Each VOI was separated into integral (Int), cortical (Cort), and trabecular (Trab) compartments for which bone mass (Mass) and volume (Vol) were determined. For the FH, however, only integral volume was measured. Cortical thickness (CortThick) of each VOI was also measured. Further, the FH and FN VOIs were each divided into four quadrants to assess the differential volume responses of their superior, inferior, posterior and anterior parts. The FN cross-sectional area (FNCSA) was calculated by the FN VOI Int volume/neck VOI height, The MIAF TH VOI was calculated as the sum of the FN, TR and IT VOIs (25). The details of measurements by MIAF-Femur have been described previously (20, 22). Precision and accuracy outcomes of MIAF-Femur have been reported earlier (20, 23). Further, to assess the internal distribution of bone, we proposed a geometric measure of the ratio of Cort/TrabMass (cortical/trabecular bone mass) of femur VOIs, which represents the cortex instability. Since in the intertrochanteric VOI, cortical bone contributes to most of the bone mass of the whole VOI, we did not calculate the ratio of Cort/TrabMass for the intertrochanteric VOI.

### Statistics

Continuous variables were reported as mean ± standard deviation (SD). The Shapiro-Wilk test was used to evaluate data for normality. Covariance Analysis (ANCOVA) was used to examine group differences for normally distributed variables. The Mann-Whitney test was used for non-normal variables. A generalized linear model (GLM) with adjustment for age, height and weight was used to compare differences in hip geometry and other variables between hip fracture patients and controls. Logistic regression was used to identify variables contributing to hip fractures based on the significantly different hip geometric parameters from GLM. We found that the ratios of cortical/trabecular mass of VOIs (total hip, neck and trochanter) and cortical thickness of neck, supero-anterior neck and intertrochanter were not normally distributed. Then we checked the log transformed data of these variables by P-P plots to see whether they were closer to being normally distributed. All variables were standardized to have a distribution with a mean of 0 and an SD of 1 to calculate odds ratios of fracture per SD decrease, similar to the analysis used in the EFFECT study papers (11, 26). The area under the receiver operating characteristic curve (AUC) was used as the performance characteristic. All statistical analyses were performed using IBM SPSS Statistics for Windows version 20.0 (IBM SPSS Inc., Chicago, IL). A p-value < 0.05 was considered statistically significant.

## Results

### Participants’ Characteristics

The average ages of the control and hip fracture groups were 71.7 and 72.0 years, respectively. The hip fracture patients had lower weight and higher height. More details of the characteristics of the two cohorts are shown in **Table 1**.

**Table 1:** Characteristics of participants.

Variable/VOI	SubVOI	Controls (N=326)	Hip Fractures (N=236)	P
**Age (years)**		71.7 ± 7.4	72.0 ± 8.5	0.334
**Height (cm)**		155.30 ± 18.2	157.6 ± 15.5	0.044
**Weight (kg)**		60.5 ± 11.7	57.1 ± 16.7	0.014
**Total Femur**				
	THCortVol (cm^3^)	16.1 ± 2.7	15.2 ± 2.6	<0.001
	THCortThick (mm)	1.9 ± 0.2	1.8 ± 0.2	<0.001
	RTHCTM	3.0 ± 1.2 (1.3^*^)	4.2 ± 2.4 (2.0^*^)	0.006
**Femoral Head**				
	HeadVol (cm^3^)	35.7 ± 5.5	37.9 ± 5.9	<0.001
	HeadVol_IP (cm^3^)	9.4 ± 1.7	9.5 ± 1.8	0.594
	HeadVol_IA (cm^3^)	9.9 ± 1.7	10.1 ± 1.9	0.175
	HeadVol_SP (cm^3^)	8.1 ± 1.6	8.9 ± 1.8	<0.001
	HeadVol_SA (cm^3^)	8.4 ± 1.6	9.4 ± 2	<0.001
**Femoral Neck**				
	FNboxVol (cm^3^)	3.7 ± 1.4	3.2 ± 0.5	<0.001
	FNCSA (cm^2^)	7.4 ± 2.8	6.4 ± 0.9	<0.001
	FNCortVol (cm^3^)	4.5 ± 1.1	4.2 ± 1.1	<0.001
	RFNCTM	3.2 ± 1.8 (1.7^*^)	4.3 ± 3.3 (2.3^*^)	<0.001
	FNCortVol_IP (cm^3^)	1.3 ± 0.4	1.2 ± 0.4	<0.001
	FNCortVol_SP (cm^3^)	1 ± 0.3	0.9 ± 0.2	<0.001
	FNCortVol_IA (cm^3^)	1.2 ± 0.3	1.1 ± 0.3	<0.001
	FNCortVol_SA (cm^3^)	1 ± 0.3	1 ± 0.3	0.635
	FNCortThick (mm)	1.8 ± 0.3 (0.3^*^)	1.7 ± 0.3 (0.3^*^)	<0.001
	FNCortThick_IP (mm)	2.2 ± 0.4	2 ± 0.4	<0.001
	FNCortThick_SP (mm)	1.8 ± 0.3	1.7 ± 0.3	<0.001
	FNCortThick_IA (mm)	1.7 ± 0.3	1.5 ± 0.3	<0.001
	FNCortThick_SA (mm)	1.6 ± 0.3 (0.3^*^)	1.5 ± 0.4 (0.4^*^)	<0.001
**Trochanter**				
	TRCortVol (cm^3^)	6.2 ± 1.2	6.1 ± 1.1	0.163
	TRCortThick (mm)	1.9 ± 0.3	1.7 ± 0.2	<0.001
	RTRCTM	2.9 ± 1.3 (1.4^*^)	3.9 ± 1.8 (1.8^*^)	<0.001
**Intertrochanter**				
	ITCortVol (cm^3^)	5.3 ± 1.5	4.9 ± 1.3	0.001
	ITCortThick (mm)	2.1 ± 0.3 (0.4^*^)	1.9 ± 0.2 (0.3^*^)	<0.001

TH, total hip; VOI, volume of interest; Vol, volume; Cort, cortical; Thick, thickness; CortThick, cortical thickness; HeadVol, femoral head volume; RTHCTM, ratio of total hip cortical/trabecular mass; RFNCTM, ratio of femoral neck cortical/trabecular mass; RTRCTM, ratio of trochanter cortical/trabecular mass; FNCSA, femoral neck cross-sectional area; TR Trochanter; IT, intertrachanter; SA, Supero-anterior; IA, Infero-anterior; IP, Infero-posterior; SP, Supero-posterior.

P values represent the comparison outcomes of Covariance Analysis (ANCOVA) for normally distributed variables and the Mann-Whitney test for non-normal variables.

^*^Refers to the interquartile range (IQR) for the non-normal variables.

### Cortical Volume and Thickness

The hip fracture cohort had significantly lower CortVol and CortThick in all VOIs except for TRVol. In the fracture cohort, the ratio of cortical to total bone mass was significantly higher for the TH, FN and TR VOIs. A closer inspection of the quadrants showed that at the FN, in the fracture patients, CortVol and CortThick were significantly lower in all quadrants except for CortVol of quadrant SA (p = 0.635). Details are summarized in **Table 1**.

### Femoral Head and Neck Volume

**Figure 2:**
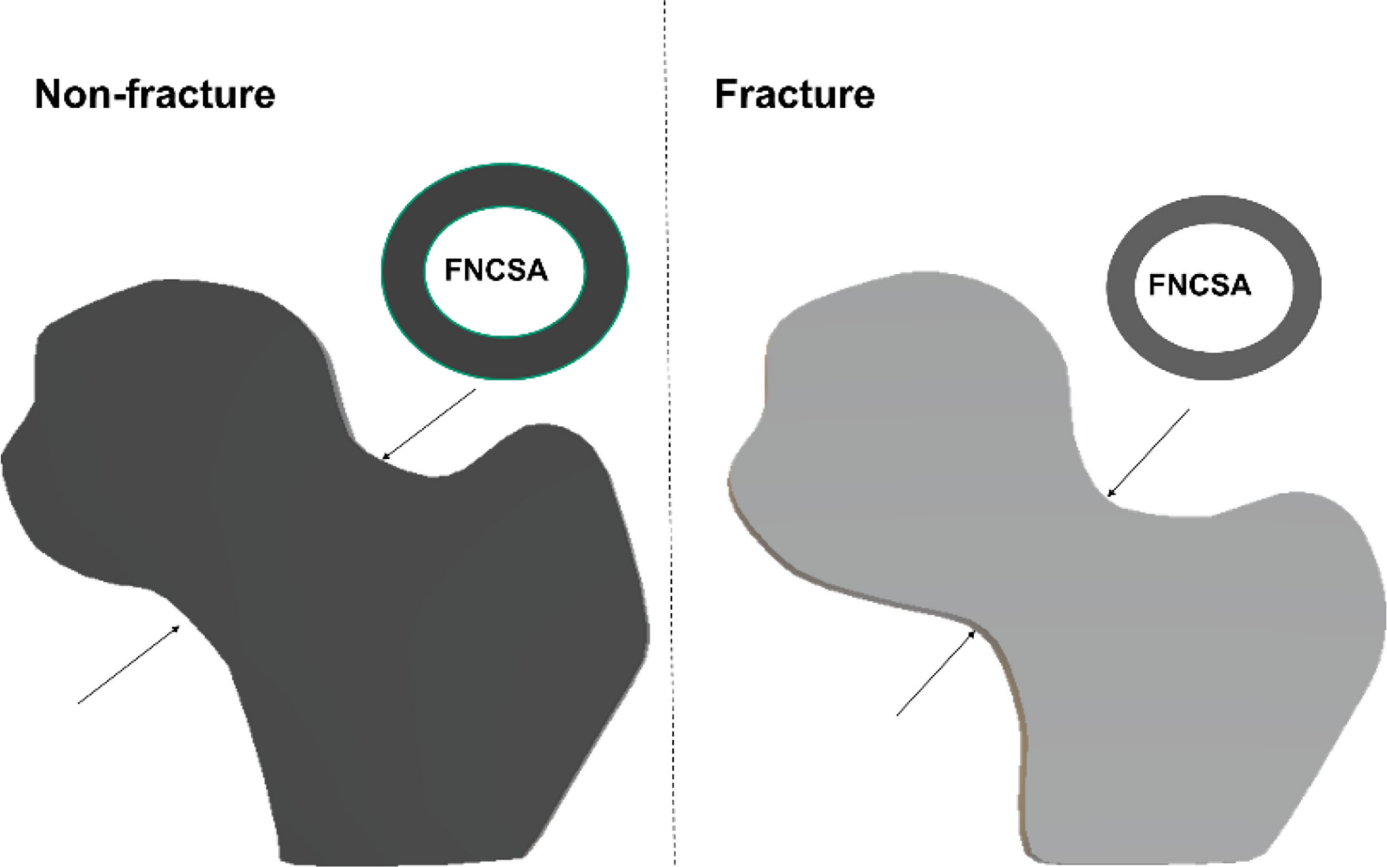
Simple conceptual impression of femoral head volume and femoral neck cross-sectional area (FNCSA) indicates hip fracture subjects with larger head volume [adjusted odd ratio (OR) 1.57; 95% CI 1.30-1.90] and smaller FNCSA (adjusted OR 1.68; 95% CI 1.35-2.10) after adjustment of age, height and weight.

Femoral head volume of the entire FH and the superior quadrants was higher (p < 0.05 for quadrants SP and SA) in the hip fracture cohort. However, the hip fracture patients had smaller integral femoral neck volume and cross-sectional area before and after adjustment for age, height and weight (**Table 1** and **Figure 2**).

### Associations of Geometry Parameters With Hip Fracture

**Figure 3:**
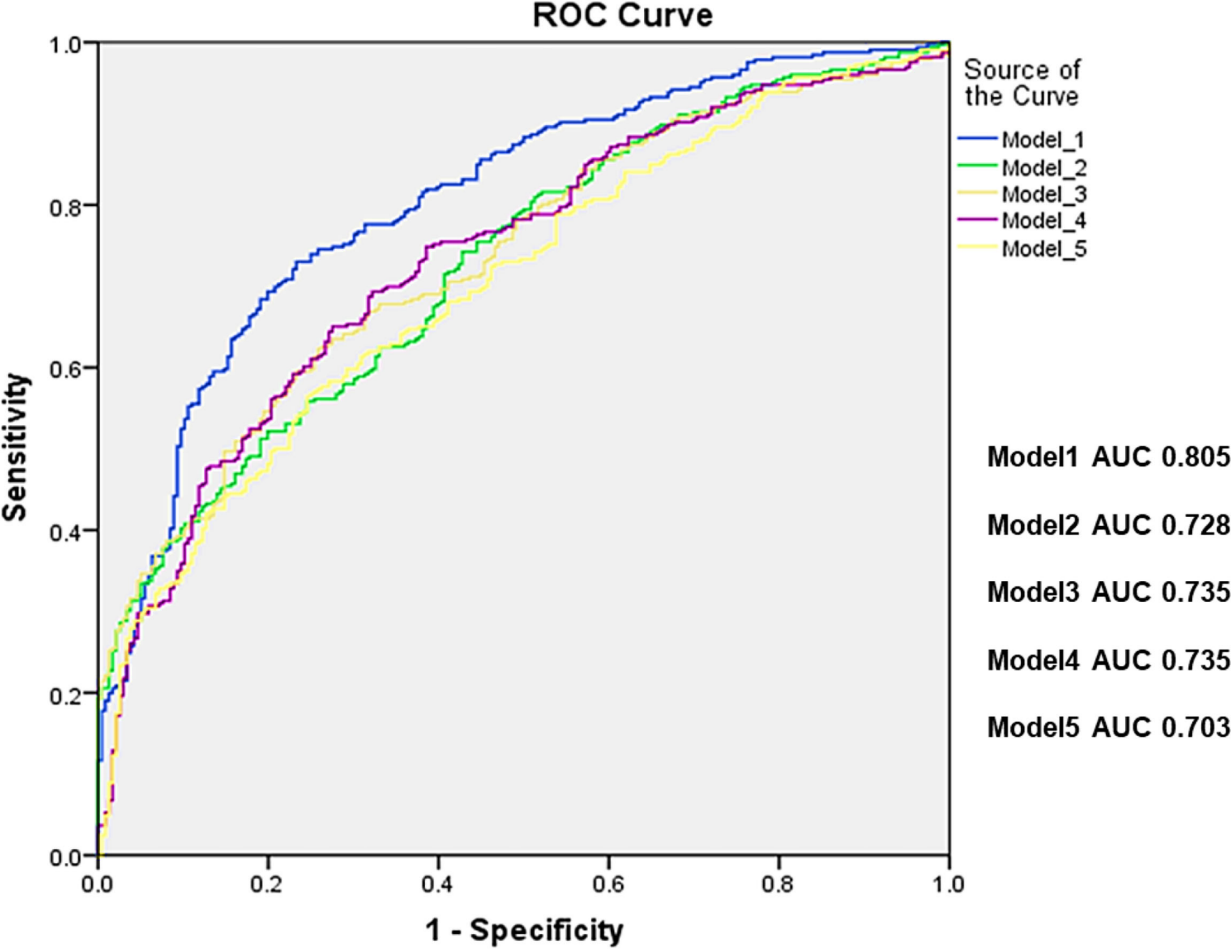
Receiver operating characteristic curves (ROC) for standard models alone (Model 1: THCortThick +HeadVol+THRCTM +FNCSA, Model 2: THCortThick+HeadVol+FNCSA, Model 3: ITCortThick+HeadVol+FNCSA, Model 4: ITCortThick+HeadVol, and model 5: ITCortThick+FNCSA. All 5 models were adjusted for age, height and weight, respectively, and the p values for all models were <0.001. TH, total hip; CortThick, cortical thickness; HeadVol, femoral head volume; RTHCTM, ratio of total hip Cortical/Trabecular Mass; FNCSA, femoral neck cross-sectional area; IT, intertrachanter.

**Table 2** shows the associations of cortical parameters with hip fractures after adjustment for age, height and weight. With respect to hip fracture discrimination, cortical volume was a poorer parameter than cortical thickness across the entire proximal femur. Amongst the cortical thickness and volume parameters, the parameter with the best discrimination was IT CortThick (odds ratio (OR) 2.10; CI 95% 1.70-2.60). The ratio of Cort/TrabMass, a measure of the internal distribution of bone, was superior to cortical thickness at discriminating hip fracture risk for the TH, FN, and TR VOIs (**Table 2**). The ratio of Cort/TrabMass of total hip (THRCTM) had the best discrimination amongst all the geometric variables (OR 2.57; CI 95% 1.94-3.40). Association with fracture was also determined for five selected models (Models 1–5) combining different geometric parameters. The highest AUC value of 0.805 was obtained for Model 1 (THCortThick + FHVol + THRCTM + FNCSA), and AUC values for Models 2-5 were all lower (AUC values: 0.735 to 0.703) (**Figure 3**). We repeated the GLM analysis using log transformed variables (ratios of cortical/trabecular mass of VOIs (total hip, femoral neck and trochanter) and cortical thickness of femoral neck, SA_FN and IT) and confirmed that there were still statistically significant differences between hip fracture patients and controls.

**Table 2:** Associations of cortical volume and thickness with hip fracture.

Cortical Variables	Unadj.OR	95%CI		Adj.OR	95%CI	
**THCortVol**	1.44	1.20	1.74	1.39	1.15	1.67
**THCortThick**	2.00	1.63	2.45	1.93	1.57	2.37
**FNCortVol**	1.39	1.17	1.66	1.37	1.15	1.64
**FNCortVol_IP**	1.50	1.26	1.80	1.47	1.22	1.77
**FNCortVol_SP**	1.43	1.20	1.70	1.42	1.19	1.71
**FNCortVol_IA**	1.44	1.21	1.72	1.43	1.19	1.72
**FNCortThick**	1.77	1.45	2.15	1.71	1.40	2.09
**FNCortThick_IP**	1.58	1.31	1.89	1.49	1.24	1.80
**FNCortThick_SP**	1.62	1.34	1.95	1.59	1.31	1.93
**FNCortThick_IA**	1.76	1.44	2.14	1.75	1.43	2.15
**FNCortThick_SA**	1.40	1.17	1.69	1.39	1.16	1.68
**TRCortVol**	1.13	0.95	1.34	1.08	0.90	1.29
**TRCortThick**	1.60	1.32	1.93	1.56	1.29	1.89
**ITCortVol**	1.36	1.13	1.62	1.32	1.10	1.58
**ITCortThick**	2.18	1.77	2.70	2.10	1.70	2.60
**RTHCTM**	2.48	1.90	3.22	2.57	1.94	3.40
**RFNCTM**	1.71	1.35	2.17	1.70	1.34	2.16
**RTRCTM**	2.07	1.67	2.55	2.08	1.67	2.60

Adjusted for age, height and weight. Unadj., unadjusted; Adj., adjusted; OR, odd ratio; TH, total hip; VOI, volume of interest; Vol, volume; Cort, cortical; Thick, thickness; CortThick, cortical thickness; FN, Femoral neck; HeadVol, femoral head volume; Int, integral; RTHCTM, ratio of total hip cortical/trabecular mass; RFNCTM, ratio of femoral neck cortical/trabecular mass; RTRCTM, ratio of trochanter cortical/trabecular mass; FNCSA, femoral neck cross-sectional area; TR, Trochanter; IT, intertrachanter; SA, Supero-anterior; IA, Infero-anterior; IP, Infero-posterior; SP, Supero-posterior.

## Discussion

Based on the analysis of 562 participants enrolled in the CASH cross-sectional case–control cohort, our study shows structural differences between elderly women with and without hip fractures, and a combination of selected geometry variables resulted in equivalent discrimination power to the aBMD model reported previously (11, 24). Our study outcomes also confirm observations of previous studies that the addition of bone volume did not significantly improve hip fracture discrimination. However, inclusion of the FH volume may allow improved prediction of hip fracture propensity.

An interesting finding of this study was that elderly women with hip fracture had larger FH but smaller FN size compared to controls. The femoral head connects continuously with the femoral neck. Thus, the head directly participates in the weight-bearing transfer to the femoral neck and the femoral neck and the trochanter are affected by the stresses and strains in the femoral head (23). Therefore, with respect to hip fracture risk prediction, the traditional DXA regions such as the FN, TR and IT may not be fully adequate to capture the risk of hip fracture. However, up to now, only two studies have reported the relationship between bone deterioration of the femoral head and hip fractures. In the European Femur Fracture Study (EFFECT) the femoral head BMD was associated with hip fracture but there was no difference in femoral head volume between participants with and without hip fracture (11). In the other QCT study, loss of FH vBMD was also found to be related to hip fracture (27). Our findings demonstrated that femoral head volume discriminated hip fracture risk with an AUC value of 0.67 after adjusting for age, height and weight, and inclusion of the FH volume improved the power of the model (**Figure 3**). Associations between geometric features of the proximal femur and hip fracture have been extensively investigated. For example, the strength of the femur is associated with the shape and size of its cross sections, the lengths of its neck and shaft, the neck–shaft angle, etc. (12). Differences in geometry of the proximal femur between women with and without hip fracture (larger head but a smaller neck in fractured subjects) identified by our study offer a new view of the femur strength and may prove useful in the construction of finite element models.

In agreement with three previous QCT studies (11, 13, 26), our results confirmed that with respect to hip fracture discrimination, cortical volume is an inferior parameter compared to cortical thickness. Previous studies have shown the power of cortical bone in resisting fracture and in hip fracture risk prediction (10, 13, 14, 26, 28–31), although the accurate measurement of cortical bone is still challenging due to the partial volume effect (25). One BMD combined with one geometry variable, for example TR vBMD with one structural parameter (e.g. FN cortical thickness), would be the preferred method of discriminating hip fracture risk using hip QCT (12, 26). The ratio of cortical/trabecular bone mass, a measure of the internal distribution of bone, is a superior parameter to cortical thickness in discriminating hip fracture risk across the entire proximal femur.

The combination of selected geometry variables in this study resulted in a similar AUC value (0.805) as the use of aBMD alone (AUC 0.796 or 0.804) reported previously in case-control studies (11, 24). Further, the AUC values of the combination of selected geometry variables in this study were similar to those reported for reference aBMD in prospective studies, ranging from 0.70 to 0.86 (32–38). Although AUC and OR results varied amongst these studies of different datasets, evidence is accumulating for a slight improvement in hip fracture risk assessment. The resulting five best-subset models for discrimination of hip fractures are ordered according to the BIC information criterion of the best-subset procedure, which combines number of variables and goodness of fit of the binary regression model (26). Similar to an earlier study (12), the combination of BMD measures and geometric parameters improved association with hip fracture but results have to be validated in prospective cohort studies. Unfortunately, the radiation dose of QCT scans hampers the application in osteoporosis screening and frailty hip fracture risk assessments. The integration of QCT-based geometry evaluations may trigger a paradigm shift in hip fracture prediction, namely, under certain circumstances, such geometry parameters could be derived from clinical routine CT images and used as predictors of hip fracture risk.

Our study has several limitations. First, due to the cross-sectional design, the analysis was limited to the evaluation of associations with hip fracture instead of prediction. Second, our results were confined to Chinese women, although our findings are consistent with a few Caucasian studies (11, 39). Third, we did not include comparisons with BMD measurements but only focused on geometric parameters. Fourth, we only studied the intact contralateral femur of the hip fracture patients by taking advantage of the anatomical similarity with the fractured side (40) despite the fact that some subjects hips can be surprisingly asymmetric.

In conclusion, there are substantial differences in total and cortical volume as well as cortical thickness between women with and without hip fractures across the entire proximal femur. The combination of geometric variables resulted in similar discrimination power for hip fracture risk as aBMD alone.

## Data Availability Statement

The original contributions presented in the study are included in the article/supplementary material. Further inquiries can be directed to the corresponding authors.

## Ethics Statement

The studies involving human participants were reviewed and approved by Beijing Jishuitan Hospital. The patients/participants provided their written informed consent to participate in this study.

## Author Contributions

XW and XC had full access to the data, take responsibility for the content, and guarantee the integrity and accuracy of the work undertaken. LW, MY, XW, KE, and XC designed the study. LW and YL led the analysis with input from MY, YG, SZ, KE, and GB. YL and YS did the literature search. YS, YG, and SZ collected the data. YL did the measurements. All authors contributed to data interpretation. LW and YL wrote the manuscript and all authors reviewed the manuscript.

## Funding

This work is supported in part by Beijing Hospitals Authority Clinical Medicine Development of Special Funding Support (code: ZYLX202107), Beijing Hospitals Authority Youth Programme (code: QMS20200402), and the National Natural Science Foundation of China (Grant Nos. 81901718, 81771831,82072445).

## Conflict of Interest

KE is a part-time employee of BioClinica, Inc.

The remaining authors declare that the research was conducted in the absence of any commercial or financial relationships that could be construed as a potential conflict of interest.

## Publisher’s Note

All claims expressed in this article are solely those of the authors and do not necessarily represent those of their affiliated organizations, or those of the publisher, the editors and the reviewers. Any product that may be evaluated in this article, or claim that may be made by its manufacturer, is not guaranteed or endorsed by the publisher.
